# Cohort Profile: The Lebanon Study on Aging and HeAlth (LSAHA)

**DOI:** 10.21203/rs.3.rs-7697794/v1

**Published:** 2025-09-25

**Authors:** Carlos Mendes de Leon, Martine Elbejjani, Sawsan Abdulrahim, Halla Ghattas, Aya El Sammak, Alexandra Abi Nassif, Frederic Conrad, Raphael Nishimura, Stephen J. McCall, Abla M. Sibai, Monique Chaaya

**Affiliations:** Georgetown University School of Health; American University of Beirut; American University of Beirut; University of South Carolina; American University of Beirut; American University of Beirut; University of Michigan; University of Michigan; American University of Beirut; American University of Beirut; American University of Beirut

**Keywords:** Alzheimer’s disease, dementia, cohort studies, aging, war exposures, life-course risk factors

## Abstract

**Background:**

This paper describes the design and cohort profile of the Lebanon Study on Aging and HeAlth (LSAHA), the first population-level study of Alzheimer’s Disease and Related Dementias (ADRD) in an Arab country. The burden due to ADRD in the Middle East and North Africa (MENA) region is among the highest in the world, but reliable population-level data on ADRD in the region are lacking. Older adults in Lebanon have experienced prolonged periods of social and economic instability due to political conflicts and chronic government mismanagement. The effects of these destabilizing experiences throughout the life course on ADRD risk and other late-life health outcomes are currently unknown.

**Methods:**

LSAHA is designed as a prospective cohort study of ADRD in a large sample of adults aged ≥ 60 years in Lebanon. We employed a probability-based multi-stage sampling design in two pre-selected areas, the city of Beirut and district of Zahle, to represent the full range of urban-rural, socio-economic, and religious diversity in Lebanon. Data collection included a standardized survey questionnaire including validated cognitive tests and anthropometric measurements, a household interview, key informant assessments, and a blood sample. Survey weights were computed to account for differential non-response and calibrated for the age- and sex-distribution in the two study areas.

**Results:**

LSAHA enrolled 3,027 participants, 1,510 from Beirut and 1,517 from Zahle, realizing a response rate of 69%. The average age of the sample was 71.7 years and 55.3% was female, 43.1% had primary education or less, while 19.9% had university training. There was a high prevalence of chronic medical conditions, such as hypertension (57.2%), heart disease (32.4%), and diabetes (32.7%). There was also a high prevalence of moderate/severe symptoms of depression (51.4%) and anxiety (34.1%). A substantial percentage reported fair or poor self-rated memory (52.1%) or having worse memory compared to 2 years ago (38.4%).

**Conclusions:**

We successfully launched a new cohort study of older adults in Lebanon to investigate ADRD and its risk factors. Data from this study will inform clinical care and policy goals in Lebanon and other settings that face a rapidly growing number of older adults with ADRD.

## BACKGROUND

This paper describes the design and cohort profile of the Lebanon Study on Aging and HeAlth (LSAHA). LSAHA is the first large-scale, population-level study of Alzheimer’s Disease and Related Dementias (ADRD) and other aging-related health challenges in the Middle East and North Africa (MENA) region. The social and political circumstances in Lebanon over the last few decades render a systematic investigation of ADRD risk and other aging-related health conditions particularly relevant. Adults who are currently aging in Lebanon have experienced prolonged exposure to intense social and political upheaval throughout much of their life course—a civil war from 1975 to 1990, internal displacement, foreign occupation, recurring episodes of political instability, and chronic government mismanagement [1, 2]. These conditions have taxed the social and economic stability of the country, which was further aggravated by the severe economic crisis of 2019 that saw a significant portion of the population descend into poverty [3], the port blast in August 2020, and renewed hostilities in southern Lebanon and Beirut that started in October, 2023. Examining the effects of such destabilizing, stressful experiences throughout the life course on ADRD risk and other late-life health outcomes is a primary objective of the study.

There is a dearth of systematic population-level data on the magnitude of ADRD risk in Lebanon and Arab populations more generally. According to data from the Global Burden of Disease Study, the burden due to ADRD in the MENA region is among the highest globally, and especially Lebanon is projected to experience among the largest increases in ADRD in the next 25 years [4]. These projections reflect the growing burden due to ADRD that will disproportionally affect low- and middle-income countries (LMIC) in future decades [5, 6]. Insights from LSAHA on ADRD and its primary risk factors will therefore likely have relevance for ADRD risk in other LMIC, especially those that have gone through similar periods of social, political, and economic instability.

An important reason for the absence of population-level data on ADRD in Lebanon is the lack of well-validated, comprehensive measures of cognitive functioning and dementia outcomes that can be administered in large samples of older adults [7]. The few previous epidemiologic studies of dementia in the Arab region have relied primarily on short screening tests to identify likely ADRD cases in the general population [8–10]. An exception is a study of 502 older adults in Lebanon that relied on the 10/66 cognitive battery [11]. In LSAHA, we used the approach of the Harmonized Cognitive Assessment Protocol (HCAP) as the basis of our cognitive battery, which is being implemented in about a dozen studies globally [12, 13]. Using the HCAP model provides a comprehensive cognitive assessment among older adults in Lebanon and enables international comparisons regarding the prevalence and incidence of ADRD and its most important risk factors across different populations in the future.

The main objectives of LSAHA are to investigate contextually informed risk factors for ADRD in the Lebanese population, and to provide critical new data on aging-related health and care needs of older adults living with ADRD and their families. The design of LSAHA was aimed at establishing a cohort of adults aged 60 years and older that would be generally representative of the older adult population in Lebanon and of sufficient size to produce statistically valid and robust findings. It follows the general methodology of the Health and Retirement Study (HRS) in the U.S. [14], which has now been adopted in about 50 large-scale population studies of older adult populations in both high-income countries (HIC) and LMIC around the globe. These studies are connected with one another in the HRS Around the World Network [15], and many of these studies also include an HCAP for the assessment of cognitive functioning and ADRD.

## METHODS

### Study population

The LSAHA study population was defined as older adults aged 60 years and over living in Lebanon. Implementing a nation-wide probability sampling strategy was not feasible due to the lack of a census or other administrative records to serve as a population listing. Moreover, security conditions limited access to certain areas of the country and made a true nation-wide sampling strategy impractical. Instead, we a priori selected two geographic areas for our sampling plan: the city of Beirut located in the governorate of Beirut and the district of Zahle located in the governorate of Beqaa. Together, these areas are meant to capture the full range of urban-rural, socio-economic, and religious diversity that characterizes the entire population of Lebanon. The two areas were also expected to incorporate the full spectrum of the primary exposures of interest, in particular those related to the prolonged periods of political conflict and violence and the recent economic crisis and hardships.

### Sampling design

LSAHA employed a probability-based multi-stage sampling design in the two pre-selected areas (strata) with a target sample size of 3,000, equally allocated by stratum, with the goal of recruiting 1,500 participants in each. We relied on gridded-population datasets created by WorldPop to provide estimates of population size within a uniform grid size [16]. Both strata were comprised of administrative areas, including areas where lack of local cooperation or security concerns would prohibit any field operations, which were, therefore, removed from the sampling frame. Beirut includes 13 administrative areas of which two areas were excluded for lack of residential population. Next, we created square-shape grids of 100 × 100 meters, or groups of such grids across the entire city, which were then proportionately allocated across the 11 remaining administrative areas. We then selected a simple random sample of grids from each administrative area to serve as primary sampling unit (PSU). In the Zahle stratum, we stratified the 58 administrative areas implicitly according to religion and socio-economic status and then selected the 25 administrative areas as PSUs using probability proportional to estimated size (PPeS), in which the estimated size was obtained by combining estimates provided by mayors of municipalities and WorldPop population estimates. We proportionately allocated grids across the 25 selected PSUs and selected a random sample of grids or groups of grids in each PSU as the secondary sampling unit (SSU). We created maps of all selected grids and sequentially numbered all buildings within each grid. Enumerators made up to three attempts to contact all residential units in each building, except those deemed to be unoccupied or abandoned. After contact with a unit was made, a household roster form was completed with an adult member of the household. We selected up to two age-eligible adults from each household; in households with more than two age-eligible adults, two adults were randomly selected with equal probability using a computer algorithm. Other than age, the inclusion criteria were ability to speak Arabic, having resided in the household for at least three months during the past year, and having no plans to leave Lebanon during the next six months.

### Sampling weights

We computed sampling weights based on the inverse of the probability of selection at all stages of the sampling design. These weights were subsequently adjusted to account for differential non-response at the SSU and housing unit levels. Finally, the weights were calibrated by age and sex according to population estimates provided by the Central Administration for Statistics – Lebanon [17].

#### Ethical approval and consent

All selected adults were invited to participate in the study and asked to provide written informed consent. Participants were asked to complete a brief decision competency test (DCT), which assesses their understanding of the study objectives and the scope and nature of the study assessments. Participants who did not demonstrate adequate understanding on this assessment (N = 159) were considered ineligible. All recruitment and assessment procedures adhered to the Helsinki Declaration guidelines and were reviewed and approved by the ethical review board of the American University of Beirut (AUB) in Beirut, Lebanon.

### Data collection (Interviewing)

Data collection included the following elements: a household interview with a member of the selected household as the participant (this could be the participant him/herself), an interview with the participant including anthropometric assessments (individual interview), an interview with a key informant of the participant (informant interview), and a blood draw. Recruitment and data collection for the participant interview started on October 9, 2023 and was completed on September 10, 2024. Data collection from the key informants was completed on September 30, 2024, and collection of blood samples was completed on April 10, 2025.

The individual interview involved the administration of a two-hour computer-assisted interview, that included a broad range of standardized tests and questions covering various domains such as demographic characteristics, social and economic conditions including retirement and pensions, physical and mental health, and war exposures (described below in further detail). If a participant elected to complete the interview via a proxy respondent, that proxy was asked to complete a shortened version of the interview without any of the cognitive or physical tests.

The key informant interview, completed via phone by someone designated by the participant, included standardized measures of the participant’s daily functioning and memory status. The household interview covered aspects of the physical conditions of the home environment, the financial assets and expenditures of the household, as well as questions about food and water security.

All participants were invited to donate a blood sample which was usually collected several weeks after the individual interview. Blood samples were immediately analyzed for several clinically relevant biomarkers (e.g., cholesterol, HbA1c) that were shared with the participant, while the remainder of the samples were aliquoted and stored in a biobank for future analysis.

### Measures & instruments (What has been measured)

The study questionnaire was developed to comprehensively capture health, social, economic, and lifestyle indicators relevant to aging and to incorporate contextual factors that are particularly important in the Lebanese setting, including economic hardships, exposure to war and conflict, and care needs arrangements. The selection of individual measures, scales and tests was informed by the availability of measures and tests previously used and validated in Lebanon and those used in HRS and its network of studies around the world. Cognitive tests were selected to align with HCAP assessments used in other studies. The final selection of questionnaire measures was based on generating inventories of instruments from HRS studies (with an emphasis on those in LMICs and comparable settings to Lebanon) and prior studies in Lebanon. Measurement alignment with the HRS and HCAP networks is intended to facilitate data harmonization and international comparative research in the future. Measures obtained across all the study components are summarized in Table 1.

### Individual Interview

The individual interview was composed of the following sections:Socio-demographic section: This included standardized questions on age, sex, marital status, nationality/country of origin, language and multilingualism, education, parental consanguinity, childhood socioeconomic status, work status, main lifetime occupation and retirement.

Cognitive assessment section: A systematic search was conducted to identify validated cognitive tests among Arabic-speaking older adults [7]. The core of the cognitive assessment was based on the 10/66 Dementia Research Group (DRG) diagnostic assessment battery [18], which was previously validated for use in older Lebanese adults [19]. The 10/66 battery includes items assessing orientation in time and space, memory functions (immediate and delayed recall of the Consortium to Establish a Registry for Alzheimer’s Disease (CERAD) word list learning task and immediate story recall), the animal naming test to assess verbal fluency, and the Community Screening Instrument for Dementia (CSI’D) scale with items for verbal fluency and constructional praxis [20]. The battery also includes the Euro-D (depression) scale, which assesses depression-like symptoms in the general population [21]. To enhance the comprehensiveness of our cognitive assessment and informed by our literature search and the HCAP mapping of cognitive tests to cognitive domains, additional tests were included to enrich the assessment of the memory, executive function, and verbal fluency domains. We added a test of delayed memory (story recall complementing the immediate story recall part of the 10/66); three tests of executive functioning: the Symbol Cancellation Test [22], the Symbol Digital Modality Test [23], two abstraction/similarities questions from the MoCA (Montreal Cognitive Assessment, Arabic validation); and a letter fluency test. We tested these cognitive tests in a series of cognitive interviews to examine their validity for administration to older adults in Lebanon [24].

Health status section: This included standardized questions regarding self-reported history of physician-diagnosed chronic medical conditions (e.g., hypertension, diabetes, cardiovascular disease), age at diagnosis and treatment. Other questions focused on smoking (cigarette and water-pipe), alcohol consumption, reproductive health, hearing and eyesight, sleep, pain symptoms, functional limitations and self-care disabilities, and access to health care. In addition, we included a COVID-19 module, and two measures of mental health, the 8-item Center for Epidemiological Studies-Depression scale (CESD-8)[25, 26] and the 7-item Generalized Anxiety Disorder (GAD-7)[27]. We used previously validated cutoff scores of 9 on the CESD-8 [28] and of 10 on the GAD-7 [27] to classify moderate to severe symptom levels on each measure.

Physical and anthropometric measurement section: This included assessment of blood pressure and pulse rate (three times); measurement of weight, height, and knee height; grip strength; and physical performance tests of gait speed and tandem stand [29, 30].

War exposures section: This section included standardized questions on experiences of physical harm and losses (home, investment properties, job) to oneself and among immediate family members [31].

Social networks and support section: This section included questions about contacts with social network members, including children, other close family members, and friends. Availability of social support was assessed using selected items from the Modified Medical Outcomes Study Social Support Survey (mMOS-SS) [32] and questions about the family or friend relationships that provide the most emotional support.

Neighborhood characteristics section: This section included 6 questions about the neighborhood environment, such as perceived safety and degree of integration into the neighborhood.

### Key Informant Interview

This interview was composed of several instruments to evaluate the cognitive and functional status of the respondent as perceived by a key informant, including the Informant Questionnaire on Cognitive Decline among the Elderly (IQCODE) [33], the Blessed Dementia Scale [34], and the Informant Community Screening Interview for Dementia (IF-CSI’D) [20].

### Household Interview

This interview included standardized questions on the household infrastructure (e.g., electricity, generators), income and assets, expenditures, food and water insecurity, and the impact of the economic crisis on the household.

### Blood draw

This involved the draw of a 22 ml venous fasting blood sample, including 5 ml of serum collected using serum separator tubes (SST), and 13,5 ml plasma and 3,5 ml whole blood for DNA abstraction, both using EDTA tubes. Blood samples were immediately transported to a laboratory at AUB for further processing and storage in a biobank. Part of the samples were also immediately analyzed for common clinical biomarkers of disease (e.g., cholesterol, HbA1c). The results of these tests were shared with the participants.

## RESULTS

**Figure 1:**
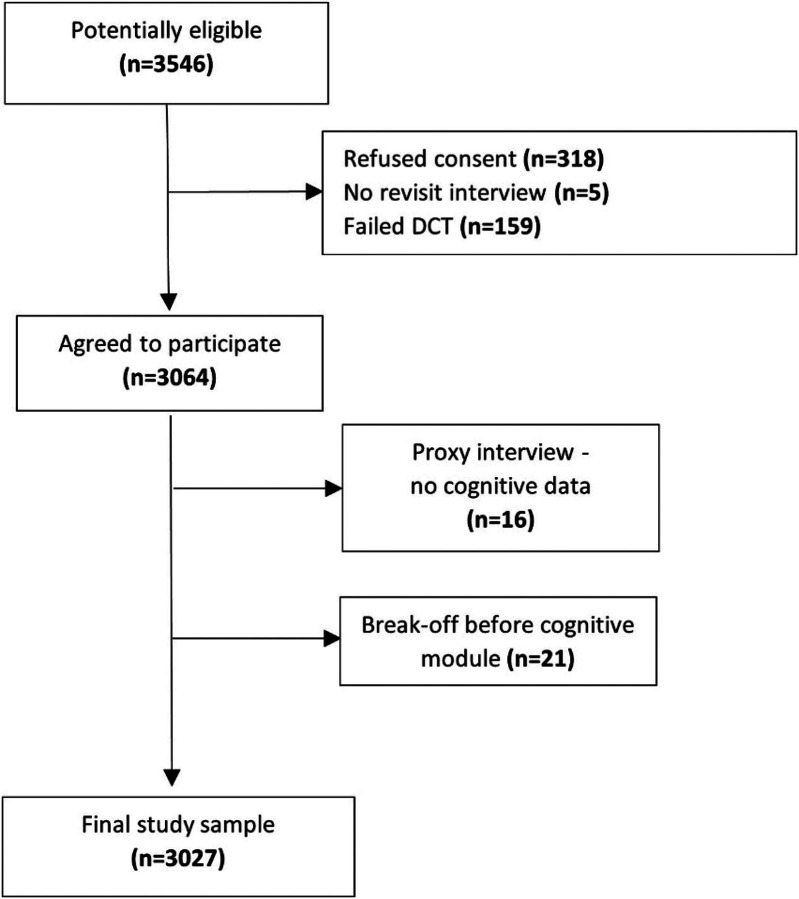
LSAHA sampling tree

LSAHA identified a total of 3,546 potentially eligible participants, of whom 318 refused to participate, 5 we were unable to schedule a revisit, and 159 did not pass the decision competency test. Of the remaining 3,064 participants, 16 elected to have a proxy interview and 21 stopped the interview before reaching the section with cognitive tests. This left a final sample size of 3,027 participants who completed the cognitive test battery section (see Fig. 1 – sampling diagram). The total response rate was 69 percent, using standard definitions from the American Association for Public Opinion Research [35], after accounting for refusals and potentially eligible participants in households we were unable to screen. The response rate was 65 percent in Beirut and 78 percent in Zahle. We collected the Informant Questionnaire for 2373 participants (78%), and the Household Questionnaire for 1997 (66%) participants. A total of 2392 (79%) of the participants donated blood samples, of which 2264 (75%) were stored in a biobank.

The basic demographic and socio-economic variables are presented in Table 2. The average age of the sample was 71.7 (SD 8.1), with almost half the sample being between 60 and 69 years old while 20% was 80 or older. Although almost two-thirds of the participants were female (n = 1904), they accounted for 55.3 percent after applying the sampling weights. About two-thirds (65.1%) of the participants were married, and 21.6% were widowed. The large majority was of Lebanese origin, with 5.3 percent being of Syrian origin. Over 40% (43.1%) of the sample had attained primary education or less, and one in five (19.9%) had received at least some university training. The average number of assets in a household, out of 10 assets assessed, was 6.7 (SD 2.1). Close to 1 out of 10 (8.8%) of the participants reported having 3 assets or less, while more than half (59.7%) reported having 7 or more of these assets. Slightly more than 10 percent (12.3%) were still working, about a quarter were retired (26.7%), while the remainder were either not currently working or had never worked.

The sample from Beirut was slightly older, more likely to be female, while the Zahle sample was more likely of Syrian origin. The Beirut sample also had higher levels of education, more assets, and was more likely to be retired, while the Zahle sample was more likely to not be working or have never worked (66.7%).

A summary of health- and cognition-related characteristics is presented in Table 3. Almost half the sample (48.7%) were current smokers, and three-quarters were either overweight (34.6%) or obese (37.7%). Hypertension (57.2%) and high cholesterol (49.0%) were the most prevalent chronic health conditions, followed by diabetes (32.7%) and heart disease (32.4%). About half (51.4%) and a third (34.1%) reported moderate to severe symptoms of depression and anxiety, respectively. Almost one in ten (9.1%) of the participants reported difficulties in at least one ADL, and one in seven (13.9%) reported difficulties in at least one IADL. More than two-thirds of the sample reported to be in fair (48.1%) or poor (18.7%) self-rated health.

The health profiles were fairly similar, although the Beirut sample reported a somewhat higher prevalence of high cholesterol, heart disease, diabetes, cancer and psychiatric conditions, possibly reflecting more access to the health care system. In contrast, the Zahle sample reported a considerably higher prevalence of moderate to severe symptoms of depression and anxiety, ADL or IADL disability, and poor subjective health.

In terms of markers of cognitive functioning, over half the sample (52.1%) reported having fair or poor memory while only about 1 in 6 (16.0%) reported having excellent or very good memory. About half (49.8%) also reported having worse or much worse memory compared to 2 years ago while 45.3 percent described their memory to be about the same as two years ago. Data from informants indicated a decline in mental health functioning in 7.6 percent and difficulties in remembering things in 30.4 percent of the participants. Self-rated and informant-rated evaluations of memory and mental health functioning were considerably lower in the Zahle sample than the Beirut sample.

## DISCUSSION

LSAHA represents the first large-scale epidemiological study in the Arab region that includes a comprehensive, validated battery to characterize cognitive functioning and identify cases of ADRD in the general older adult population. LSAHA will address the paucity of robust epidemiologic data from well-designed population studies in the MENA region (El Metwally, 2019) which is projected to face some of the highest rates of increase in ADRD globally [4]. Most existing data on ADRD in the Arab region come from a few cross-sectional surveys, mostly in Egypt, relying primarily on short screening instruments to identify ADRD cases, or on hospital-based case series on nursing homes residents [7, 8]. Building on a previous pilot study of about 500 older adults in Lebanon [11, 36], LSAHA is designed as a longitudinal study to provide critically new information on the prevalence and incidence of ADRD, with a comprehensive and well-validated battery of cognitive tests and a large sample size to produce sufficient power to establish the role of the primary risk factors in this population. Our objective is not only to examine the role of established ADRD risk factors, such as age, educational attainment and chronic medical conditions including hypertension and diabetes, but also to investigate novel risk factors that are relevant in this setting, in particular exposure to political instability and violence and its economic ramifications during prolonged periods of the life course.

We achieved our recruitment goal of 3,000 participants aged ≥ 60 years, enrolling a total of 3,027 participants. We faced two important challenges in developing a sampling design that would yield a representative sample of the older adult population in Lebanon, mainly due to the lack of a population census and the security situation in certain areas of the country. Moreover, data collection in the field started on October 9, 2023, two days after the start of the conflict in Gaza. This required us to make further adjustments to the sampling strategy, as additional areas that originally were deemed safe were no longer available for the sampling plan. We also invested increased efforts to inform political and security stakeholders and conducted general media and local community outreach activities to announce the study in sampled study areas. To maximize diversity, our sampling plan focused on two purposefully selected study areas in Beirut and Zahle. Our final sample shows ample variability in the basic socio-demographic and health-related characteristics, providing confidence that we obtained a sample that is likely to be reasonably representative of the overall older adult population in Lebanon.

We found a high level of poor mental health in the sample, with a high prevalence of moderate to severe symptoms of depression and anxiety. These symptom levels fall in the upper range of what has been reported for other LMIC populations [37, 38], while being substantially lower in HIC populations [37, 39–41]. It seems likely that the renewed hostilities in Lebanon in the fall of 2023, coinciding with the start of our data collection, and the impact of the severe economic crisis of 2019 that left many older adults significantly impoverished, may have contributed to these findings. Both these factors are well-established determinants of poor mental health [42–46].

An important strength of LSAHA is that it has adopted the rigorous survey methods of the around 50 studies in the HRS Around The World Network [15]. LSAHA has also implemented the detailed assessment of multiple cognitive function domains as developed by HCAP in order to produce reliable ascertainment of ADRD cases in the general population [12, 13] and to provide a comprehensive assessment of baseline cognitive functioning for future studies of cognitive changes and decline. Through these international networks, we will be able to participate in comparative analyses regarding the epidemiology of ADRD across the globe. A particularly exciting opportunity in this regard relates to LSAHA’s unique focus on the role of prolonged exposure to political conflict and violence in dementia risk. This feature will enable us to identify contextually relevant risk factors and compare them with data on ADRD risk factors from other populations, particularly those with older generations that have experienced similar exposures in their life course, such as those in Northern Ireland [47] and Vietnam [48].

An important goal for LSAHA is to share these data with fellow scientists and stakeholders in Lebanon and around the world. A curated dataset will be made available on the Gateway to Global Aging platform (https://g2aging.org/home) in the near future. LSAHA data are expected to inform several immediate and long-term clinical care and policy goals in Lebanon and across the MENA region. Clinically, the validated cognitive test battery assessing multiple domains may provide the basis for new screening methods that can be integrated in health care settings to identify older adults at risk of cognitive decline and dementia. They will also contribute to a better understanding of the primary risk factors for dementia in this population, which may serve as a basis for screening and prevention strategies. From a policy perspective, LSAHA data will add to the evidence base for a national dementia strategy that will serve to guide workforce training, resource allocations and development of appropriate interventions for vulnerable and at-risk subgroups in the population. Finally, new information on ADRD risks associated with stressful exposures throughout the life course may be useful to the development of screening and prevention policies in other LMICs facing similar demographic, social, and political challenges.

## Figures and Tables

**Table 1:** Summary of measures collected in Lebanon Study on Ageing and HeAlth (LSAHA)

Forms	Measures
Individual interview	Current and childhood sociodemographic characteristics, work and retirement, cognitive assessments, physical health, mental health, sleep, cigarette and waterpipe smoking, alcohol consumption, functional limitations, healthcare utilization, war exposure, caregiving and unmet needs, social network and support, neighborhood characteristics
Physical measurements	Blood pressure, weight, height, knee length, grip strength, walking speed, balance test
Blood sample collection	Total cholesterol, high-density lipoprotein cholesterol, low-density lipoprotein cholesterol, thyroid stimulating hormone, glycated hemoglobin, C-reactive protein, vitamin D. Storage of samples at −80°C.
Household interview (any household member ≥ 18 years)	Living arrangements, household characteristics, assets and services, household consumption and income, food and water security, economic crisis impact
Informant questionnaire	Informant socioeconomic status, IQCODE, Blessed dementia rating scale, Informant CSI’D

**Table 2:** Lebanon Study on Aging and HeAlth (LSAHA) – sociodemographic variables

	Total	Beirut	Zahle
Characteristics	Mean (SD) or N (%)*	Mean (SD) or N (%)*	Mean (SD) or N (%)*
**Participants (n)** *	3027	1510	1517
**Age (years)**	71.7 (8.1)	72.1 (8.2)	70.7 (8.0)
**Age**			
60–69	1602 (47.4)	763 (45.4)	839 (53.2)
70–79	984 (32.6)	522 (32.8)	462 (32.1)
>80	441 (20.0)	225 (21.8)	216 (14.8)
**Sex**			
Female	1904 (55.3)	931 (56.2)	973 (52.5)
Male	1123 (44.7)	579 (43.8)	544 (47.5)
**Marital status**			
Married	1805 (65.1)	911 (64.7)	894 (66.1)
Widowed	803 (21.6)	368 (20.6)	435 (24.4)
Divorced/separated	132 (4.3)	85 (5.0)	47 (2.2)
Never married	284 (9.1)	146 (9.8)	138 (7.3)
**Nationality**			
Lebanese	2738 (91.5)	1401 (93.3)	1337 (86.6)
Syrian	167 (5.3)	66 (4.2)	101 (8.4)
Other	122 (3.2)	43 (2.6)	79 (5.0)
**Education**			
None/primary	1430 (43.1)	584 (37.1)	846 (60.3)
Intermediate/secondary	1123 (37.1)	593 (38.9)	530 (31.9)
University degree	456 (19.9)	328 (24.0)	128 (7.8)
**Number of assets** **	6.7 (2.1)	7.0 (1.9)	5.8 (2.3)
**Number of assets (categories)** **			
0–3	237 (8.8)	64 (5.2)	173 (18.1)
4–6	914 (31.5)	395 (28.7)	519 (38.8)
7+	1396 (59.7)	776 (66.1)	620 (43.1)
**Work status**			
Currently working	296 (12.3)	187 (13.0)	109 (10.1)
Retired	680 (26.7)	390 (28.0)	290 (23.2)
Not working or never worked	1992 (61.0)	919 (59.1)	1073 (66.7)

* Percentages are weighted to produce population estimates

** Sum of the following 10 assets: functional water heater, washing machine, satellite dish or cable, microwave, dishwasher, air conditioner, oven, refrigerator, car or truck, and access to internet.

**Table 3:** Lebanon Study on Aging and HeAlth (LSAHA) – health-related variables

	Total	Beirut	Zahle
Characteristics	Mean (SD) or N (%)*	Mean (SD) or N (%)*	Mean (SD) or N (%)*
Participants (n)*	3027	1510	1517
**Smoking status**			
Never	934 (29.6)	388 (28.1)	546 (34.0)
Past	559 (21.7)	309 (22.8)	250 (18.4)
Current	1409 (48.7)	750 (49.1)	659 (47.7)
**Body mass index (BMI)**			
Underweight (BMI <18)	34 (1.5)	16 (1.5)	18 (1.5)
Normal (BMI 18–25)	496 (26.3)	259 (26.9)	237 (24.4)
Overweight (BMI 25–30)	741 (34.6)	379 (34.8)	362 (33.8)
Obese (BMI>30)	823 (37.7)	424 (36.7)	399 (40.3)
**Medical conditions**			
Hypertension	1662 (57.2)	835 (56.9)	827 (58.0)
High cholesterol	1338 (49.0)	748 (51.2)	590 (42.4)
Heart disease	880 (32.4)	481 (33.3)	399 (29.9)
Diabetes	921 (32.7)	499 (33.7)	422 (29.8)
Arthritis	565 (19.8)	297 (20.1)	268 (18.9)
Cancer	178 (7.6)	99 (8.5)	79 (5.0)
Psychiatric conditions	364 (12.6)	207 (13.6)	157 (9.7)
**Moderate/severe depression** **	1545 (51.4)	715 (49.2)	830 (57.7)
**Moderate/severe anxiety** ***	1061 (34.1)	499 (31.3)	562 (42.5)
**≥ 1 ADL difficulties**	271 (9.1)	123 (8.1)	148 (11.8)
**≥ 1 IADL difficulties**	406 (13.9)	196 (12.8)	210 (17.1)
**Self-rated health**			
Excellent/very good	246 (8.4)	126 (8.9)	120 (7.0)
Good	657 (24.8)	349 (25.7)	308 (22.1)
Fair	1409 (48.1)	710 (48.4)	699 (47.2)
Poor	595 (18.7)	261 (17.0)	334 (23.6)
**Self-rated memory at the present time**			
Excellent	167 (5.9)	79 (5.9)	88 (5.8)
Very good	278 (10.1)	151 (10.4)	127 (9.3)
Good	891 (32.0)	474 (33.1)	417 (28.8)
Fair	1278 (43.9)	623 (42.6)	655 (47.7)
Poor	289 (8.2)	118 (8.1)	171 (8.5)
**Self-rated memory compared to two years ago**			
Much better now	69 (2.4)	35 (2.3)	34 (2.8)
Better now	82 (2.5)	34 (2.1)	48 (3.7)
About the same	1245 (45.3)	689 (48.4)	556 (36.2)
Worse now	1141 (38.4)	523 (36.3)	618 (44.5)
Much worse now than it was then	365 (11.4)	163 (10.8)	202 (12.9)
**Informant-rated decline in mental functioning**			
No change	2092 (92.4)	1056 (94.8)	1036 (85.8)
General decline	227 (7.6)	65 (5.2)	162 (14.2)
**Informant-rated difficulties in remembering things**			
No	1556 (69.6)	803 (72.5)	753 (61.8)
Yes	736 (30.4)	308 (27.5)	428 (38.2)

* Percentages are weighted to produce population estimates

** Score ≥ 9 on the 8-item Centre for Epidemiologic Studies Depression Scale (CES-D 8)

*** Score ≥ 10 on the Generalized Anxiety Disorder-7 test (GAD-7)

## Data Availability

The datasets used and/or analysed during the current study are available from the corresponding author on reasonable request.

## Abbreviations

LSAHA: Lebanon Study on Aging and HeAlth
ADRD: Alzheimer’s Disease and Related Dementias
MENA: Middle East and North Africa
LMIC: Low- and middle-income countries
HCAP: Harmonized Cognitive Assessment Protocol
HRS: Health and Retirement Study
HIC: High-Income Countries
PSU: Primary Sampling Unit
PPeS: Proportional to Estimated Size
SSU: Secondary Sampling Unit
AUB: American University of Beirut
DRG: Dementia Research Group
CERAD: Consortium to Establish a Registry for Alzheimer’s Disease
CSI’D: Community Screening Instrument for Dementia
MoCA: Montreal Cognitive Assessment
IQCODE: Informant Questionnaire on Cognitive Decline among the Elderly
IF-CSI’D: Informant Community Screening Interview for Dementia
SST: Serum Separator Tubes
CESD-8: 8-item Center for Epidemiological Studies Depression Scale
GAD7: 7-item Generalized Anxiety Disorder
